# Whey-based diet containing medium chain triglycerides modulates the gut microbiota and protects the intestinal mucosa from chemotherapy while maintaining therapy efficacy

**DOI:** 10.1038/s41419-023-05850-9

**Published:** 2023-05-23

**Authors:** Hannah R. Wardill, Ana Rita Da Silva Ferreira, Himanshu Kumar, Emma H. Bateman, Courtney B. Cross, Joanne M. Bowen, Rick Havinga, Hermie J. M. Harmsen, Jan Knol, Bram Dorresteijn, Miriam van Dijk, Jeroen van Bergenhenegouwen, Wim J. E. Tissing

**Affiliations:** 1 Department of Pediatrics, University of Groningen, University Medical Center Groningen, Groningen, The Netherlands; 2 School of Biomedicine, Faculty of Health and Medical Sciences, The University of Adelaide, Adelaide, SA Australia; 3 Supportive Oncology Research Group, Precision Cancer Medicine, The South Australian Health and Medical Research Institute, Adelaide, Australia; 4 Department of Medical Microbiology, University of Groningen, University Medical Center Groningen, Groningen, The Netherlands; 5 Danone Nutricia Research, Utrecht, the Netherlands; 6 Laboratory of Microbiology, Wageningen University, Wageningen, The Netherlands; 7 Princess Máxima Center for Pediatric Oncology, Utrecht, The Netherlands

**Keywords:** Diarrhoea, Preclinical research, Malnutrition, Cancer models

## Abstract

Cytotoxicity (i.e. cell death) is the core mechanism by which chemotherapy induces its anti-cancer effects. Unfortunately, this same mechanism underpins the collateral damage it causes to healthy tissues. The gastrointestinal tract is highly susceptible to chemotherapy’s cytotoxicity, resulting in ulcerative lesions (termed gastrointestinal mucositis, GI-M) that impair the functional capacity of the gut leading to diarrhea, anorexia, malnutrition and weight loss, which negatively impact physical/psychological wellbeing and treatment adherence. Preventing these side effects has proven challenging given the overlapping mechanisms that dictate chemotherapy efficacy and toxicity. Here, we report on a novel dietary intervention that, due to its localized gastrointestinal effects, is able to protect the intestinal mucosal from unwanted toxicity without impairing the anti-tumor effects of chemotherapy. The test diet (containing extensively hydrolyzed whey protein and medium chain triglycerides (MCTs)), was investigated in both tumor-naïve and tumor-bearing models to evaluate its effect on GI-M and chemo-efficacy, respectively. In both models, methotrexate was used as the representative chemotherapeutic agent and the diet was provided *ad libitum* for 14 days prior to treatment. GI-M was measured using the validated biomarker plasma citrulline, and chemo-efficacy defined by tumor burden (cm^3^/g body weight). The test diet significantly attenuated GI-M (*P* = 0.03), with associated reductions in diarrhea (*P* < 0.0001), weight loss (*P* < 0.05), daily activity (*P* < 0.02) and maintenance of body composition (*P* < 0.02). Moreover, the test diet showed significant impact on gut microbiota by increasing diversity and resilience, whilst also altering microbial composition and function (indicated by cecal short and brained chain fatty acids). The test diet did not impair the efficacy of methotrexate against mammary adenocarcinoma (tumor) cells. In line with the first model, the test diet minimized intestinal injury (*P* = 0.001) and diarrhea (*P* < 0.0001). These data support translational initiatives to determine the clinical feasibility, utility and efficacy of this diet to improve chemotherapy treatment outcomes.

## Background

Despite the excitement that surrounds novel targeted agents, chemotherapy continues to be the mainstay therapy for most advanced cancers. Contrary to its longstanding use, chemotherapy continues to cause a variety of highly impactful side effects as a result of its non-selective cytotoxicity characterized by profound cell death in healthy tissues. These side effects detrimentally affect the physical and psychosocial wellbeing of patients, and their ability/willingness to receive intended dosing [1]. By and large, these side effects remain without effective intervention, owing to the difficult challenge of preventing unwanted cell death in healthy tissues without impairing desired cell death in tumors. As such, many side effects are superficially managed via the modification of chemotherapy dosing, thus increasing the risk of disease progression or relapse [1].

The gastrointestinal mucosa is highly sensitive to the non-selective cytotoxic nature of chemotherapy. Irreversible DNA damage, apoptosis and inflammatory injury to the gastrointestinal mucosa (termed “gastrointestinal mucositis”, GI-M) profoundly disrupts intestinal architecture [2], decreasing the mucosal area available for nutrient absorption [3, 4] and impairing host immune defenses [5]. Consequently, patients that develop GI-M are at a significantly higher risk of secondary complications [6], especially nutritional deficiencies. When combined with anorexia due to nausea, pain, oral dysfunction (trismus, xerostomia, ulceration, dysphagia) and taste changes, unmanageable weight loss caused by GI-M often necessitates nutritional intervention [4, 7, 8]. The change in body composition that results from malnutrition is particularly impactful, with weight loss and cachexia (muscle wasting) associated with a higher symptom burden (e.g. fatigue) and increasing the risk of death in a variety of cancer cohorts [9–12].

To date, strategies to prevent GI-M and its associated symptoms have been largely under-whelming, with current clinical practice guidelines unable to provide uniform recommendations with meaningful impacts [13]. As such, GI-M related symptoms (diarrhea, weight loss, cachexia, anorexia, fatigue) are managed reactively (e.g. enteral or parenteral nutrition) [14, 15]. This is a clinically challenging task as, if even oral intake is maintained, nutrient absorption is drastically impaired due to the profound tissue injury associated with GI-M [4]. As such, the majority of patients do not meet nutritional requirements [14, 15].

In modernizing our approach to supportive cancer care and symptom control, efforts to prophylactically intervene to minimize the severity of acute toxicities and associated symptoms must be prioritized. Numerous studies have shown that performance status and general health of the patient at the time of chemotherapy predicts the incidence and severity of treatment side effects, including GI-M [16] and weight loss/cachexia [17]. Despite this, there remain few strategies aimed at pre-habilitating a patient prior to chemotherapy [18]. When considering that 50–80% of cancer patients report involuntary weight loss at diagnosis [6], there is arguably a significant window of opportunity to provide nutritional intervention prior to chemotherapy to protect the intestinal mucosa from unwanted injury and prevent associated symptoms.

It is generally recognized that the gut microbiome plays a critical role in the protecting the integrity of the intestinal mucosa and the maintenance of mucosal homeostasis [19]. Chemotherapy has been consistently demonstrated to induce changes in the gut microbiome composition and function [20]; changes which are thought to contribute to the severity of GI-M [21]. Moreover, evidence increasingly suggests that the composition of the gut microbiome at the time of chemotherapy influences the patient’s risk of adverse toxicities [22], including GI-M. As such, dietary interventions that support a healthy microbiome before therapy and promote its resilience during/after therapy hold promise as therapeutic strategies for GI-M and its related symptoms. Here, we describe the mucoprotective and microbiota modulatory effects of an extensively hydrolyzed whey protein diet, rich in triglycerides with a medium chain (MCTs). This diet was selected based on the ability of MCTs and small peptide fragments to provide local protection to the intestinal mucosa via inhibition of aberrant inflammation [23, 24]. We hypothesized that pre-habilitation with this diet would minimize GI-M and secondary symptoms related to malnutrition, without impacting the systemic efficacy of chemotherapy.

## Materials and methods

This study is reported in line with the ARRIVE guidelines [25] for the robust and reproducible reporting of animal research. For details regarding ethical approvals and animal husbandry, please see *Supplementary Information*.

### Animal models

This study used two models of chemotherapy-induced GI-M, a tumor-naïve [26] and tumor-bearing model [27]. The tumor-naïve model was conducted at the University Medical Center Groningen (The Netherlands) and the tumor-bearing model conducted at the University of Adelaide (Australia).

#### Tumor-naïve model

To evaluate the impact of the dietary intervention on GI-M and resulting symptoms, a validated tumor-naïve model using the chemotherapeutic drug methotrexate (MTX), was used [3, 28]. Briefly, male Wistar rats (150–180 g, 6–8 weeks, Charles River Laboratories) were treated with a single dose of MTX (45 mg/kg, 50 mg/ml; obtained from Pharmachemie Holding B.V. The Netherlands) or a volume-equivalent dose of 0.9% NaCl administered intravenously via the penile vein under anesthetic (3% isoflurane) on day 0. On arrival, all rats were provided with *ad libitum* AIN93G diet (Ssniff-Spezialdiäten GmbH, Soest, Germany) for 1 week, before being randomized to receive the test (T.Diet) or control diet (C.Diet) containing the ingredients listed in Table S1/S2. These diets were provided for 2 weeks prior to MTX (i.e., starting day −14) and until termination. Rats were terminated 10 days post-MTX treatment via general isoflurane anesthesia, cardiac puncture and cervical dislocation.

The primary outcome of this model was plasma citrulline, a validated biomarker of GI-M [29] assessed as previously described [30]. Plasma citrulline concentrations on day 4 (peak GI-M) were used to perform power calculations. The study was powered to detect a relative effect of 25%, with an alpha of 0.05 and power of 0.9 (*N* = 9/group required).

#### Tumor-bearing model

To evaluate the impact of the dietary intervention on MTX efficacy, we used the well-established Dark Agouti Mammary Adenocarcinoma (DAMA) model as previously described [27]. Following acclimatization (1 week with AIN93G control diet), female DA rats weighing 150–170 g (7–9 weeks, Animal Resources Centre) were given *ad libitum* access to the test or control diet for 2 weeks prior to MTX treatment (i.e., starting day −14). On day −5 they were subcutaneously inoculated with 0.2 ml (2.0 × 10^7^ cells/ml) mammary adenocarcinoma cells (maintained in JMB’s laboratory) on each flank. MTX (2 mg/kg) or vehicle control (saline) were administered intramuscularly in two separate doses 24 h apart and rats were terminated 4 days after the first dose. Tumors were measured daily using digital callipers to determine their volume ([length*width*depth]*[π/6] expressed as cm^3^). Tumor burden was calculated as tumor volume relative to body weight (%BW, cm^3^/g).

Rats were terminated 4 days after the second MTX dose via general isoflurane anesthesia, cardiac puncture and cervical dislocation. At termination, the intestine was resected, flushed with ice-cold 1 X phosphate buffered saline (PBS, pH 7.4) weighed and processed. In addition, tumors were resected and weighed (grams) to determine a final burden relative to body weight (%BW, g/g).

### Physiological assessments and downstream analyzes

#### Body weight, diarrhea, food intake and water intake

All rats, in both models, were assessed daily for welfare (coat condition, movement, diarrhea, weight) in a room separate from where rats were housed. Rats were weighed daily (~10 am) using digital scales and change in body weight was expressed relative to the day of MTX administration (%ΔBW). Diarrhea was assessed using a previously described grading system, where 0 = no diarrhea, 1 = mild diarrhea indicated by soft but formed pellets, 2 = moderate diarrhea indicated by perianal staining of the fur and 3 = severe diarrhea indicated by abdominal staining of the fur with leakage [27]. Food and water bottles were weighed daily ( ~ 10am) to determine daily food and water intake. All assessments took ~5 min per rat.

#### Body composition

Body composition was assessed in tumor-naïve rats using the Bruker MiniSpec LF90 as per manufacturer’s guidelines. This was performed in a specialized room on day −15 (prior to diet), day −1 (prior to MTX) and day 10 (termination) to determine the amount (grams) of lean muscle mass, body fat and body fluid. Rats were restrained in a specialised tube within the Bruker MiniSpec LF90 for ~5 min. Scans were performed starting at 10am on each day following the same order based on randomization code.

#### Physical Activity

Physical activity was monitored continuously using activity sensors (dual technology detector DUO 240, Visonic; adapted by R. Visser, NIN, Amsterdam, The Netherlands) that translated individual changes in the infrared pattern caused by movements of the animals into arbitrary activity counts. Sensors were mounted above the home cages and were connected through input ports and an interface to a computer equipped with MED-PC® IV software for data collection (MED associates, St Albans, VT, USA). Activity was expressed in counts per 30 min (both for the total 24 h period, the dark period (active period) and the light period (inactive period)). Activity was calculated for each rat separately. The activities were normalized to the averaged activity during the acclimatization period to dampen the day-to-day variability [31].

#### Systemic MTX concentrations

The MTX level was quantified in plasma isolated from whole blood collected from the tail vein two days after MTX administration in tumor-naïve rats using the enzyme multiplied immunoassay (EMIT) on an automated drug analyzer (Abbott Architect C8000) [32]. MTX was analyzed in 30ul of plasma collected from *N* = 9 rats per group (pooled and run in duplicate).

#### Gut microbiome analyses

##### 16 S rRNA gene sequencing and analysis

The gut microbiome was longitudinally analyzed using 16 S rRNA gene sequencing on fecal samples collected from rats on days −15 (pre-dietary intervention), day −1 (before MTX), day +4 (peak GI-M) and day +10 (recovery). DNA extraction, PCR amplification (V3-V4 region) and 16 S rRNA gene sequencing was performed as previously described [33]. 16 S rRNA sequencing reads were demultiplexed and trimmed (*q* > 20), and pair-ends were merged using PEAR software [34]. Only merged reads were retained which fulfilled the criteria of a minimal length of 300, *q* > 25 over a window of 15 bases and no ambiguous bases. Further, reads were dereplicated and counted using mothur [35], and reads with low abundance (<2 reads over all samples) were discarded. VSEARCH was used for chimera removal [36] and RDP gold database was used as reference [37].

Deblur algorithm (which is integrated in QIIME2) was used to filter reads which contained PhiX or adapters [38, 39]. Taxonomical classification of 16 S rRNA gene was carried using the Ribosomal Database Project (RDP) classifier [40], against the SILVA_132 database. Reads with eukaryotic assignments and with low relative abundance up to 0.0005% in all samples were excluded from further downstream analysis. Moreover, samples were rarefied, and α-diversity was computed using phyloseq [41], and vegan [42] packaged in R [43].

Sequencing data was further processed for compositional analysis of microbial community using vegan package in R and statistical methods described elsewhere [44, 45]. Briefly, Shannon index was calculated for measuring alpha-diversity for each sample (using the diversity function from vegan package in R). Statistical differences at different taxonomic level were evaluated using non-parametric Mann-Whitney U tests. Distance based RDA (db-RDA) was used to assess differences in microbial community composition and bray-curtis distances were ordinated using metric scaling and the results were analyzed using redundancy analysis.

#### Quantification of short chain fatty acids

Metabolomic analyses were performed on cecal samples collected from rats at the time of termination as previously described [46]. Briefly, after being isolated, the cecum was cut at the apex and the contents expelled into a sterile tube, before being immediately snap frozen in liquid nitrogen and stored at −80oC. Samples were then thawed on ice and homogenized 1:9 in sterile milliQ water. Supernatants were isolated by centrifugation, and analyzed by GC-MS (7890 A GC System and 5975 C inert XI EI/CI MSD with an EI inert 350 source, Agilent Technologies, Santa Clara, USA).

### Statistical analyzes

All data (excluding 16 S rRNA sequencing data) were analyzed using GraphPad Prism v9.0. Data were analyzed for normality using the D’Agostino-Pearson Normality test, and when confirmed were analyzed using a one-way analysis of variance (ANOVA) or Kruskal-Wallis. For repeated measures, data were analyzed using a mixed model with Geisser’s Greenhouse correction. A Chi-squared test was used to compare diarrhea data. *P* < 0.05 was considered significant different. All statistical analyzes and descriptors of data (i.e. measure of variance) are described in figure legends. All data showed comparable variance and met assumptions for the tests employed.

## Results

This study has been reported in line with the ARRIVE guidelines for transparent preclinical reporting. There were no significant differences in baseline body weight or plasma citrulline between any of the groups in either model (Table S3).

### Hydrolyzed whey and MCT-enriched diet prevents MTX-induced GI-M and associated deconditioning in tumor-naïve rats

**Fig. 1:**
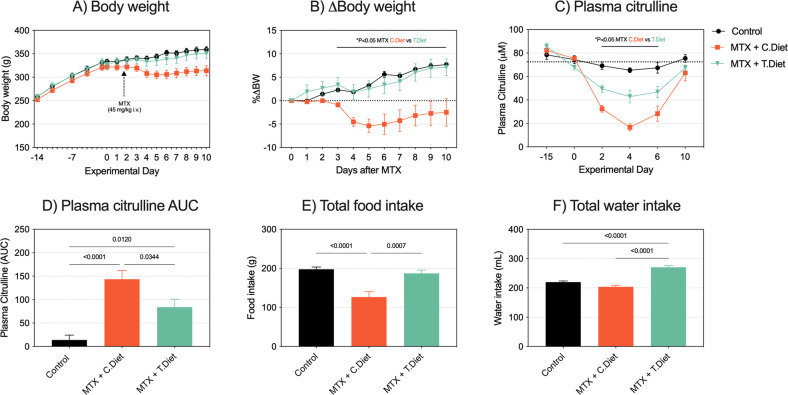
An extensively hydrolyzed whey-protein diet rich in MCTs prevents weight loss, mucosal injury and anorexia caused by methotrexate. Weight gain was comparable in rats during the induction phase, prior to MTX (**A**), with differences in weight only observed after MTX (**B**). Plasma citrulline, a biomarker of mucosal injury, decreased in both MTX treated groups (**C**), however the depth and duration were decreased in rats receiving the test diet resulting in a significantly lower AUC (**D**). Food intake, a marker of anorexia, was maintained in rats consuming the test diet (**E**). Water intake increased when consuming the test diet (**F**). All experiments were performed in *N* = 9 tumor-naïve rats treated with 45 mg/kg MTX or vehicle control. Data are shown as mean ± SEM and were analyzed using mixed models (repeated data) or a one-way ANOVA.

All rats consumed the intervention and control diet at the same rate in the 2 weeks prior to MTX treatment (Control: 20.67 ± 1.17 g/day; MTX+Control diet (C.Diet): 19.44 ± 1.49 g/day; MTX+Test diet (T.Diet): 20.93 ± 1.57 g/day). Body weights were comparable throughout this time period, with differences only observed after MTX (Fig. 1A). After MTX treatment, MTX + C.Diet rats lost a significant amount of weight compared to controls and MTX + T.Diet (Fig. 1B, *P* < 0.05 day 3–10). The test diet was also able to minimize decreases in plasma citrulline during peak GI-M (Fig. 1C), indicating less severe mucosal injury. Calculated area under the curve for longitudinal citrulline values (AUC below average of day 0 values, 72.46μm) showed an increase in MTX + C.Diet rats (Fig. 1D, *P* < 0.0001), consistent with previous data. This was decreased in MTX + T.Diet rats (*P* = 0.0344 relative to MTX + C.Diet), indicating a reduced depth and duration of mucosal injury in rats that consumed the test diet. The test diet was also able to promote alimentation, with total food intake increased compared to the MTX + C.Diet group and promote hydration (Fig. 1E, *P* = 0.0007, *P* < 0.0001, respectively).

**Fig. 2:**
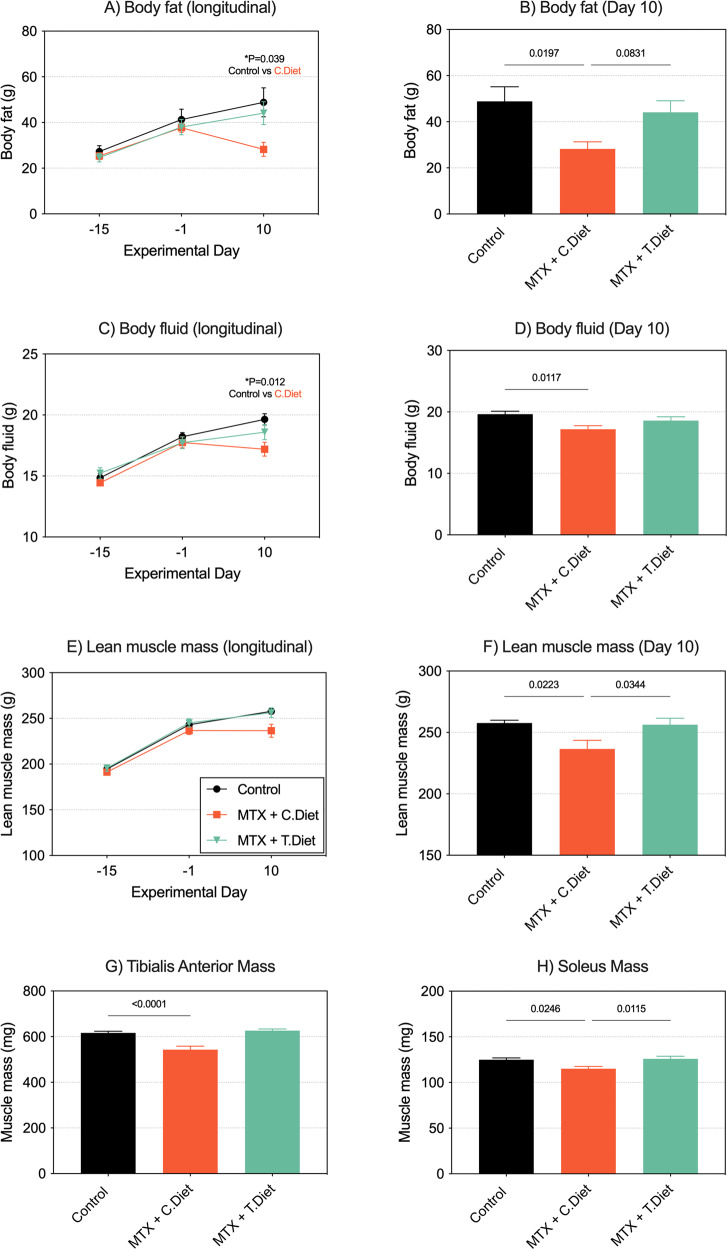
Minimizing the severity of gastrointestinal mucositis maintained body composition. Body composition was assessed longitudinally in all rats to determine body fat (**A**, **B**), body fluid (**C**, **D**) and lean muscle mass (**E**, **F**). When administered in rats consuming the control diet, MTX caused a decrease in all parameters. Consumption of the test diet mitigated these effects. At necropsy, atrophy of the tibialis anterior (**G**) and soleus (**H**) were decreased in the methotrexate+control diet group, but not the test diet group. All experiments were performed in *N* = 9 tumor-naïve rats per group. Data are shown as mean ± SEM and were analyzed using mixed models (repeated data) or a one-way ANOVA. Data are shown as mean ± SEM and were analyzed using mixed-models (repeated data) or a one-way ANOVA.

Consistent with previous reports, MTX induced mild to moderate, self-limiting diarrhea which was observed in the MTX + C.Diet group (Fig. S1, *P* < 0.0001). No episodes of diarrhea were detected for the entire experimental period for the T.Diet group. Similarly, consumption of the test diet maintained body composition, demonstrating a capacity to prevent muscle wasting and loss of fat stores. Body composition analysis in the MTX + C.Diet group confirmed that weight loss was underpinned by a decrease in body fat (Fig. 2A, B, *P* = 0.0197), body fluid (Fig. 2C, D, *P* = 0.0117) and lean muscle mass (Fig. 2E, F, *P* = 0.0223). Although unable to prevent fluid loss, the test diet maintained fat stores and lean muscle mass. This was confirmed by post-mortem analysis of the muscle *tibialis anterior* and *soleus*, both of which were decreased in the MTX + C.Diet group, but not in the MTX + T.Diet Group (Fig. 2G, H, *P* < 0.0001, *P* = 0.0246).

### Hydrolyzed whey and MCT-enriched diet promotes activity in rats treated with MTX

**Fig. 3:**
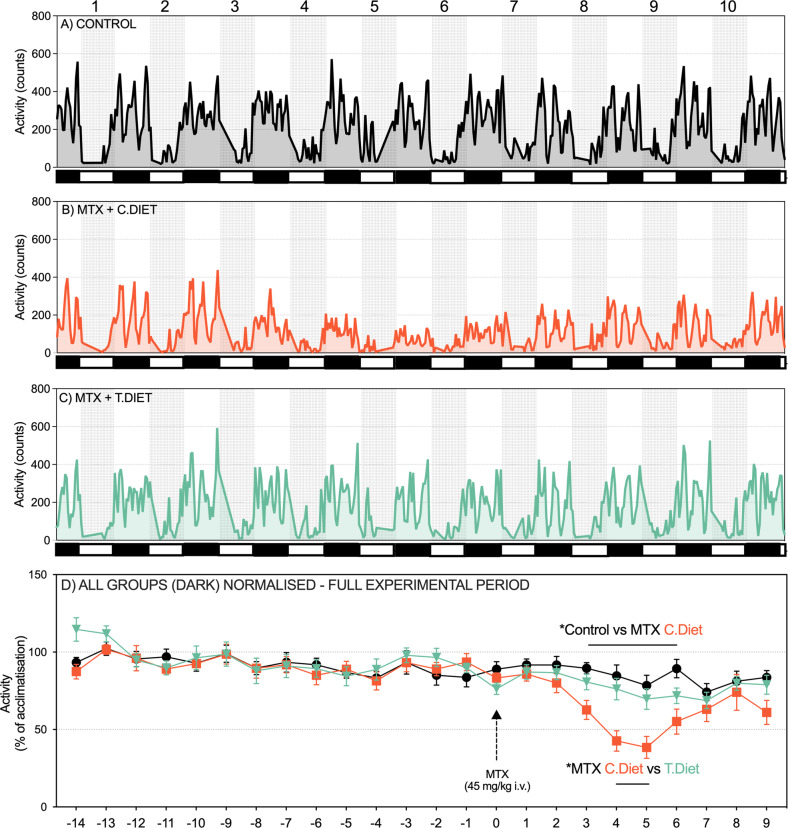
Minimizing the severity of gastrointestinal mucositis with the test diet maintained physical activity. Graphs (**A**–**C**) depict day and night activity, calculated from sensors mounted above cages connected to MED-PC® IV software collected from the day of MTX treatment. **D** shows normalized (night) activity over the entire time course, with methotrexate causing a profound decrease in daily activity from day 2–5 in the control diet. This was prevented by the test diet. All experiments were performed in *N* = 9 tumor-naïve rats per group. Data are shown as mean ± SEM and were analyzed using mixed models.

During the pre-treatment phase (day −14 to day 0), there were no differences in raw activity counts between groups during the light or dark phases (Fig. S2). In contrast, traces showed a clear decrease in activity after MTX, which was only evident in MTX + C.Diet rats. Analysis of normalized data showed comparably low activity in all groups during the light phase, however, profound differences were observed in the dark phase (Fig. 3), with a decrease in dark phase activity identified in the MTX + C.Diet group compared to both the control group (Day 3: *P* = 0.007, Day 4: *P* = 0.0014, Day 5: *P* = 0.0023, Day 6: *P* = 0.015) and the MTX + T.Diet group (Day 4: *P* = 0.0088, Day 5: *P* = 0.0163). This resulted in similar observations for total activity (i.e. dark and light phases), with the MTX + C.Diet group less active compared to the control group (Day 3: *P* = 0.0006, Day 4–6: *P* < 0.0001) and the MTX + T.Diet group (Day 3: *P* = 0.0279, Day 4: *P* < 0.0001, Day 5: *P* = 0.0004).

### The hydrolyzed whey, MCT-enriched diet modulates gut microbiome composition and function and promotes microbial resilience

**Fig. 4:**
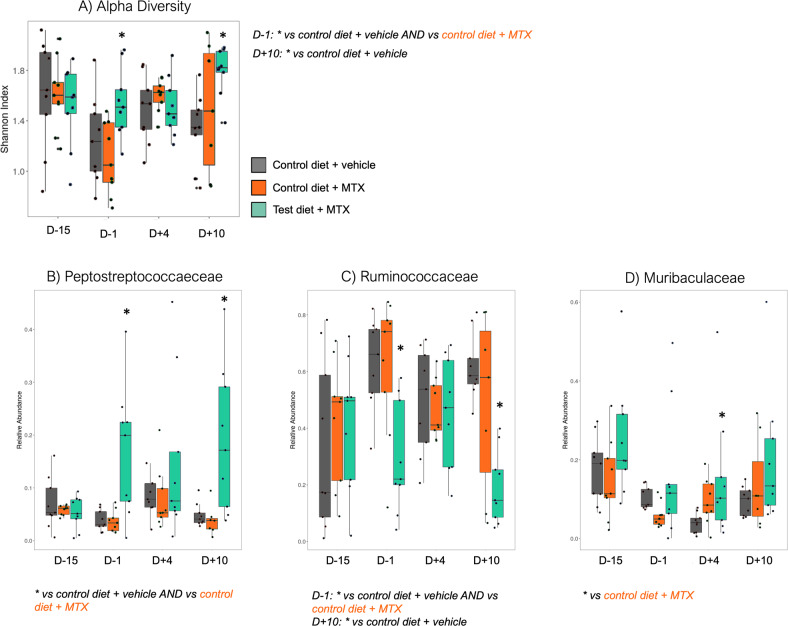
The gut (fecal) microbiome is modulated by the test diet before and after methotrexate. 16 S rRNA gene sequencing revealed an increase in alpha diversity (**A**) and altered the relative abundance of Peptostreptococcaeceae (**B**), Muribaculaceae (**C**) and Ruminococcaceae (**D**). Microbial changes were most evident at day -1 (before MTX) and day 10. All analyses were performed using repeated fecal samples collected from *N* = 9 tumor-naïve rats per group.

Gut microbiota analysis based on 16 S rRNA gene sequencing demonstrated that the test diet induced significant changes in the gut microbiome composition. Microbial diversity (as measured by Shannon’s index) was significantly higher in the test diet group compared to the vehicle control and control diet groups before MTX (Fig. 4A, *P* = 0.0503). This was accompanied by a significant compositional shift in the gut microbiome (Fig. S4A–D), associated with the expansion of the *Peptostreptococcaeceae* (Fig. 4B, *P* = 0.02) and a decrease in *Ruminococcaceae* (Fig. 4C, *P* = 0.0102). At peak GI-M (Day 4), microbial differences were limited with only a significant increase observed in *Muribaculaceae* (Fig. 4D, *P* = 0.0577). At recovery (Day 10), there was a significant rebound in Shannon’s diversity (Fig. 4A, *P* = 0.0008) and *Peptostreptococcaeceae* (Fig. 4B, *P* = 0.0124).

**Fig. 5:**
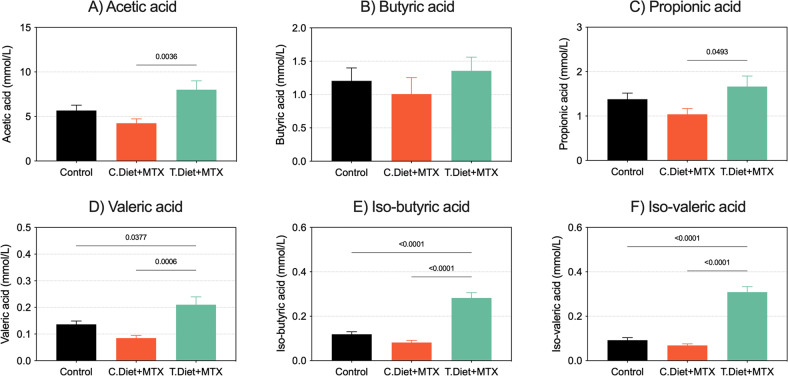
The test diet increases the production of both short- and branched-brain fatty acids. Fatty acids were analyzed in cecal contents collected at day 10. All experiments were performed in *N* = 9 tumor-naïve rats per group. Data are shown as mean ± SEM and were analyzed using mixed models.

Cecal SCFA were analyzed as a functional indicator reflecting change(s) in gut microbiota composition. MTX alone (with control diet) induced a significant decrease in *total* SCFAs (C.Diet+vehicle: 8.59 ± 2.84 mmol/L, C.Diet+MTX 6.47 ± 2.46 mmol/L, *P* = 0.002). The test diet caused a significant increase in total SCFAs compared to both the C.Diet+vehicle and C.Diet+MTX groups (11.83 ± 4.20 mmol/L, *P* < 0.0001 and *P* = 0.005, respectively). When evaluating specific SCFAs, the test diet significantly increased cecal concentrations of acetic acid (*P* = 0.003), propionic acid (*P* = 0.049), valeric acid (*P* < 0.004), iso-butyric acid (*P* < 0.0001) and iso-valeric acid (*P* < 0.0001, Fig. 5).

### MTX efficacy is not impaired by the hydrolyzed whey, MCT-enriched diet

Given the level of protection observed in our initial studies, we considered it critical to confirm that the diet was not influencing: (a) systemic levels of MTX, and (b) the anti-tumor efficacy of MTX.

**Fig. 6:**
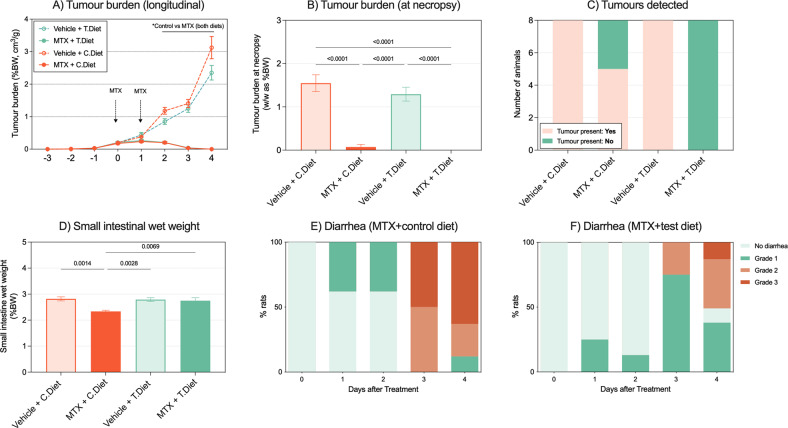
An extensively hydrolyzed protein diet enriched with MCTs does not influence tumor growth or methotrexate efficacy, whilst maintaining mucoprotective effects. Tumor burden shown longitudinally (**A**) and at necropsy (**B**) was not affected by dietary intervention. The number of tumors present at necropsy was significantly higher in the control diet compared to the test diet (**C**, *P* < 0.0001). Mucosal atrophy (**D**) and diarrhea (**E**, **F**) were minimized, but not prevented, by the test diet. All experiments were performed in *N* = 8 tumor-bearing rats per group. Data are shown as mean ± SEM and were analyzed using mixed models (**A**), a one-way ANOVA (**B**) or Chi Squared test (**C**, **E**, **F**).

Mass spectrometry confirmed there was no difference in systemic MTX concentrations across treatment groups (C.Diet: 24.5 ± 1.5μg/ml, T.Diet: 22.0 ± 0.00μg/ml, Fig. S2). Dietary intervention did not influence normal tumor growth (i.e. in the absence of MTX) nor MTX-induced tumor kill, in the tumor-bearing DAMA model (Fig. 6A). At necropsy, tumors were dissected and weighed, with no difference between MTX-treated groups (%BW, g/g, Fig. 6B). While MTX induced a profound clearance of tumors in most cases, 3 of 8 animals in the C.Diet group had visible tumors at necropsy. This was significantly higher (*P* < 0.0001, Chi squared test) than in the T.Diet group, where no rats had visible tumors (Fig. 6C).

In addition to evaluating MTX efficacy, the DAMA model also enables evaluation of GI-M and related symptoms. Small intestinal wet weight is commonly used as a surrogate marker of GI-M in this model (indicative of intestinal atrophy). MTX caused a significant decrease in intestinal wet weight in rats consuming the C.Diet (Fig. 6D, *P* = 0.0014). This was not observed in the test diet group. While diarrhea was not entirely prevented in the tumor-bearing model, the T.Diet reduced the severity of diarrhea (Fig. 6E, F, *P* < 0.0001 on Day 4). Despite these benefits, the T.Diet did not prevent weight loss or hypocitrullinaemia in the tumor-bearing model (Fig. S5).

## Discussion

Despite decades of research, chemotherapy continues to cause collateral damage to healthy tissues resulting in a range of side effects that negatively impact patient quality of life and treatment adherence. The gastrointestinal mucosa is highly susceptible to unwanted toxicity, with the resulting damage associated with high symptom burden and poor treatment outcomes. A major challenge in preventing this damage is the difficulty in targeting unwanted cell death without impairing intended cytotoxicity against the tumor. As such, many side effects including gastrointestinal mucositis (GI-M) remain poorly controlled. Here, we report on the mucoprotective and microbiome modulatory effects of a novel dietary intervention in the context of toxicity caused by the chemotherapeutic drug, methotrexate (MTX). Importantly, we show that this diet controlled a range of highly burdensome symptoms related to GI-M, but did not impair the anti-tumor efficacy of MTX. This provides a strong rationale to translate this diet to early phase clinical investigation.

The rationale for investigating this specific dietary formulation is its abundance in both extensively hydrolyzed whey protein and medium chain triglycerides (MCTs). MCTs are readily absorbed [23, 47] and provide rapid energy to promote the renewal and repair of intestinal epithelial cells, plus maintain mucosal barrier function by reinforcing tight junctions [48]. They also exert profound immunomodulatory properties [24, 47], dampening inflammation via their indirect effect on NFkB [49] – a transcription factor considered to be the gatekeeper of GI-M development [50] – and the peroxisome proliferator activated receptor (PPAR)-γ [51]. Evidence also supports MCTs stabilize the gut microbiota [52] and exert anti-microbial effects [47], impeding the growth of common pathogens (including *Salmonella* and *E. coli*) [53, 54], thus decreasing inflammatory responses mediated by pathogenic microbial products. This is an important mechanism of interest for GI-M as disruption of the gut microbiota has been documented to exacerbate GI-M via endotoxin-dependent Toll-like receptor 4 activation [21]. Furthermore, pre-therapy gut microbiota signatures have been identified to predict chemotherapy side effects [55], suggesting that dietary intervention *prior* to therapy may beneficially augment the microbiota to enhance response.

In line with their documented capacity to augment gut microbes, we observed profound changes in the gut microbiome and associated metabolome potentially induced by the test diet. In addition to an increase in microbial diversity, we observed profound expansion of the microbial taxa *Peptostreptococcaeceae* prior to MTX treatment in the test diet group. Expansion of this inflammatory-associated microbe has been reported (in offspring) in a maternal high fat diet mouse model which subsequently increased the risk of DSS-induced colitis [56]. Similarly, evidence exists to suggest that diets enriched for MCTs [57] and levels of crude dietary protein [58, 59] increase the abundance of this microbial taxa. Given that this microbial taxon is known for its contribution to proteolytic fermentation, it is likely that this microbe is expanding in response to the increased dietary protein. This results in the formation of branched chain fatty acids (iso-butyrate, iso-valeric acid) – each of which were significantly elevated in rats receiving the test diet, with paralleled increases in short chain fatty acids.

Mechanistic interpretation of these findings is complex, and it is impossible to dissect causality without further studies in which fecal samples are transferred from test diet animals to gnotobiotic (or antibiotic depleted rodents). However, it is of interest to consider that *Peptostreptococcaeceae* is known to induce inflammation. While we did not collect sufficient biospecimens to evaluate pre-MTX inflammatory markers, it could be speculated that a degree of pre-MTX inflammation may increase the host’s resilience to further inflammatory insults that drive GI-M. The paralleled increases in branched- and short-chain fatty acids (B/SCFAs) is also important to consider. In contrast to SCFAs, which have well documented effects on mucosal integrity, immune function and other mechanisms related to GI-M, BCFAs have not been well studied in the context of chemotherapy-side effects, or mucosal integrity. In the context of MTX-induced GI-M, exogenous administration of butyrate mitigated tissue damage by upregulating efflux pathways (*Abcc1*) to reduce cytotoxic drug load in enterocytes [60]. Whether this same effect is seen for iso-butyrate remains unclear and warrants further investigation. Similarly, direct effects of diet-induced microbial changes on MTX activity may be considered. Our data demonstrated no difference in systemic MTX concentrations in control and test diet groups. However, evidence shows that some microbial taxa are capable of folate biosynthesis. Folate is given exogenously to quench MTX, and as such, it is plausible that the diet prevented mucotoxicity by promoting endogenous folate production. Given we saw no detrimental effects on MTX efficacy (in our tumor-bearing model), it is plausible that this was restricted to the local intestinal environment and may offer a causal mechanism by which diet-microbe interactions mitigate MTX toxicity without impairing tumor kill. Understanding if this protection is also observed after other anti-cancer agents/therapies is also another area of future investigation, and will provide clarity on the mechanism(s) at play.

While the core focus of this study was the prevention of GI-M, given the sequalae of consequences associated with GI-M, we also aimed to determine the effect of GI-M control on broader symptom burden. As hypothesized, the test diet minimized the depth and duration of GI-M, improving the functional capacity of the mucosa to maintain nutritional demands of the host. By preserving gastrointestinal function, symptom burden was reduced. In this way, our findings may help to prompt changes in current clinical practice by encouraging *proactive* interventions aimed at pre-habilitating the patient rather than using supportive care interventions in *response* to clinical indicators. These findings also underscore the centrality of GI-M to many chemotherapy symptoms, emphasizing the broader impacts that can be achieved by preserving gastrointestinal function.

In translating new supportive care interventions to the clinic, it is critical that their potential to impact the efficacy of chemotherapy is investigated. Both the efficacy and toxicity of most chemotherapeutic drugs are governed by similar mechanisms [61], as such, there is always a risk that by controlling toxicity, treatment efficacy is compromised. We showed that systemic MTX levels were unchanged by the test diet. Furthermore, using our tumor-bearing model, we demonstrated the extensively hydrolyzed whey protein diet did not interfere with the anti-tumor efficacy of MTX. In fact, the diet appeared to enhance the efficacy of MTX, with 100% of the MTX-treated rats on the intervention diet having full tumor clearance, compared to just 62.5% of the control diet group. It is difficult to speculate the mechanisms that were responsible for this finding. However, sporadic evidence suggests that a ketogenic diet, rich in MCTs, enhances the anti-tumor efficacy of chemotherapy by inducing metabolic stress which increase cytotoxic sensitivity [62]. It is not possible to determine from this study if the tumor sensitivity was enhanced directly via MCTs and therefore suggest that this mechanism warrants further investigation.

While this study has provided critical support of MCT and extensively hydrolyzed whey protein intervention to control GI-M and resulting symptoms, it is not without its limitations. Of particular importance is the use of two different models to investigate mucoprotective effects and anti-tumor effects. It cannot be ignored that these models have been developed in different strains and sexes of rat reflecting the distinct, and differing, purposes of these already existing models. While we could have re-established these models to mitigate these differences, these models are validated for their use in evaluating anti-mucositis interventions and tumor burden, thus we decided it was most appropriate to conduct our experiments using these well-established models. Nonetheless, these differences undoubtedly influenced our results, and may be responsible for some discrepancies between the studies. For example, although we observed mucoprotective effects in the tumor-bearing DAMA model, these benefits were less apparent compared to the tumor-naïve model. In particular, it cannot be ignored that we did not observe benefits in weight loss or plasma citrulline dynamics, a biomarker of GI-M. However, the severe nature of this model, the impact of the tumor on the condition of the host and the more profound impact of single-housing on female rats (compared to group housed males in the tumor-naïve model) must be appreciated. Despite the lower MTX dose, the DAMA model is much more severe in its toxicity profile compared to the tumor-naïve model, reflecting the method of MTX administration (2 × 2 mg/kg i.m.) required to induce sufficient decreases in tumor burden. This cytotoxic injury to the tumor, as well as the physical presence of a tumor, both impact the physiology and resilience of the host and may be a significant confounding variable when considering GI-M outcomes.

It also cannot be ignored that we were unable to perform intensive evaluation of the tumor tissue given the near complete clearance of the DAMA tumor in MTX-treated rats. Mechanistically, this limits our capacity to understand *how* our dietary intervention influenced the anti-tumor effects of MTX. Understanding diet-MTX interactions therefore warrants further investigation in a model that is better suited to studying the tumor microenvironment. It is also important to note that in our attempts to prioritize longitudinal assessments and reduce the number of laboratory animals used to execute our primary objective, histopathological assessment of the gut during peak GI-M as rats were terminated at day 10 when GI-M has resolved. However, plasma citrulline is a validated biomarker shown to correlate strongly with intestinal cell death and architecture (villous height, crypt depth) [46] and we therefore believe this is a robust surrogate that negates the need to perform these assessments. This also limited our capacity to perform other tissue-based analyses to explore mechanisms of protection. Similarly, the limited amount of blood that could be collected non-terminally (~75 μl), we could not analyse systemic mediators of GI-M and other symptoms. We appreciate the interest in mechanisms that drive these responses. However, it was our priority to disseminate the benefits of this diet on outcomes that are of significant translational interest prior to characterizing the complex mechanisms by which the test diet influences GI-M and tumor clearance. Unpacking this is a focus of our ongoing research program, which will also aim to understand which dietary component is driving its efficacy, or if these are achieved by the synergistic effects of the dietary compounds.

In conclusion, we have provided the first evidence supporting benefits of early dietary intervention aimed at pre-habilitating the host, in particular the gut microbiome, to mitigate unwanted cell death in the highly vulnerable intestinal mucosa without impacting tumor kill. Our data therefore warrant further investigation to: (a) understand the mechanisms that underpin our preclinical observations, and (b) establish its clinical feasibility, utility and efficacy.

## Supplementary information

S1

S2

S3

S4

S5

Supplementary information

Reproducibility checklist

## Data Availability

All source data will be made available by corresponding author upon request, according to Nutricia Danone Research Policies.
