# Supplementary figures and images for: Whey-based diet containing medium chain triglycerides modulates the gut microbiota and protects the intestinal mucosa from chemotherapy while maintaining therapy efficacy
Source: Cell Death Dis. 2023 May 23;14(5):338. doi: 10.1038/s41419-023-05850-9 (PMC10206084; doi:10.1038/s41419-023-05850-9)

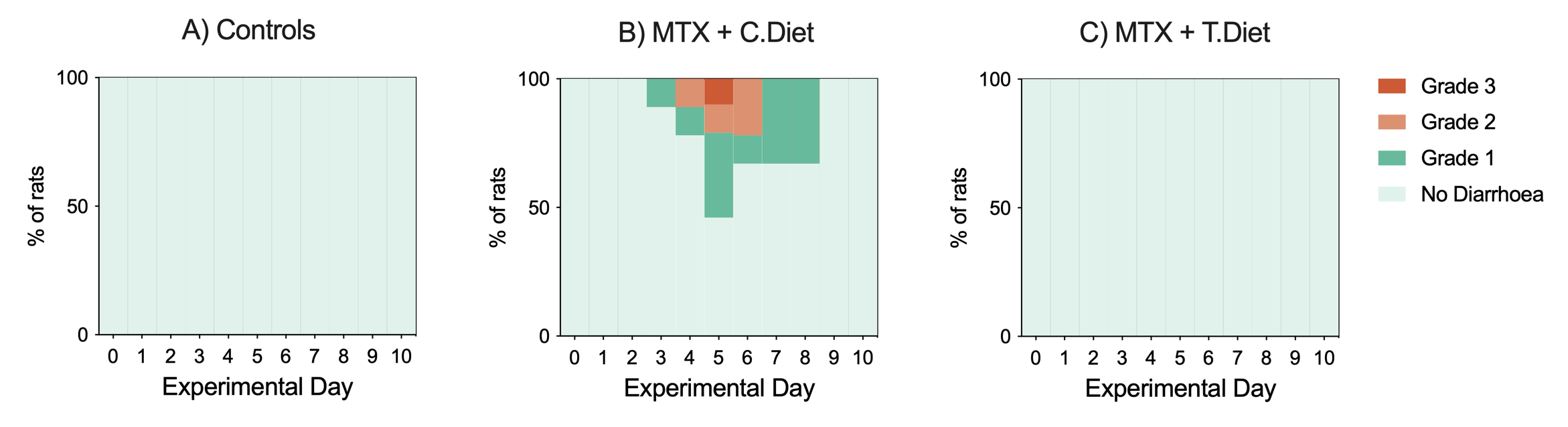

Supplement: Supplementary file 1 — S1 [file 41419_2023_5850_MOESM1_ESM.tif]

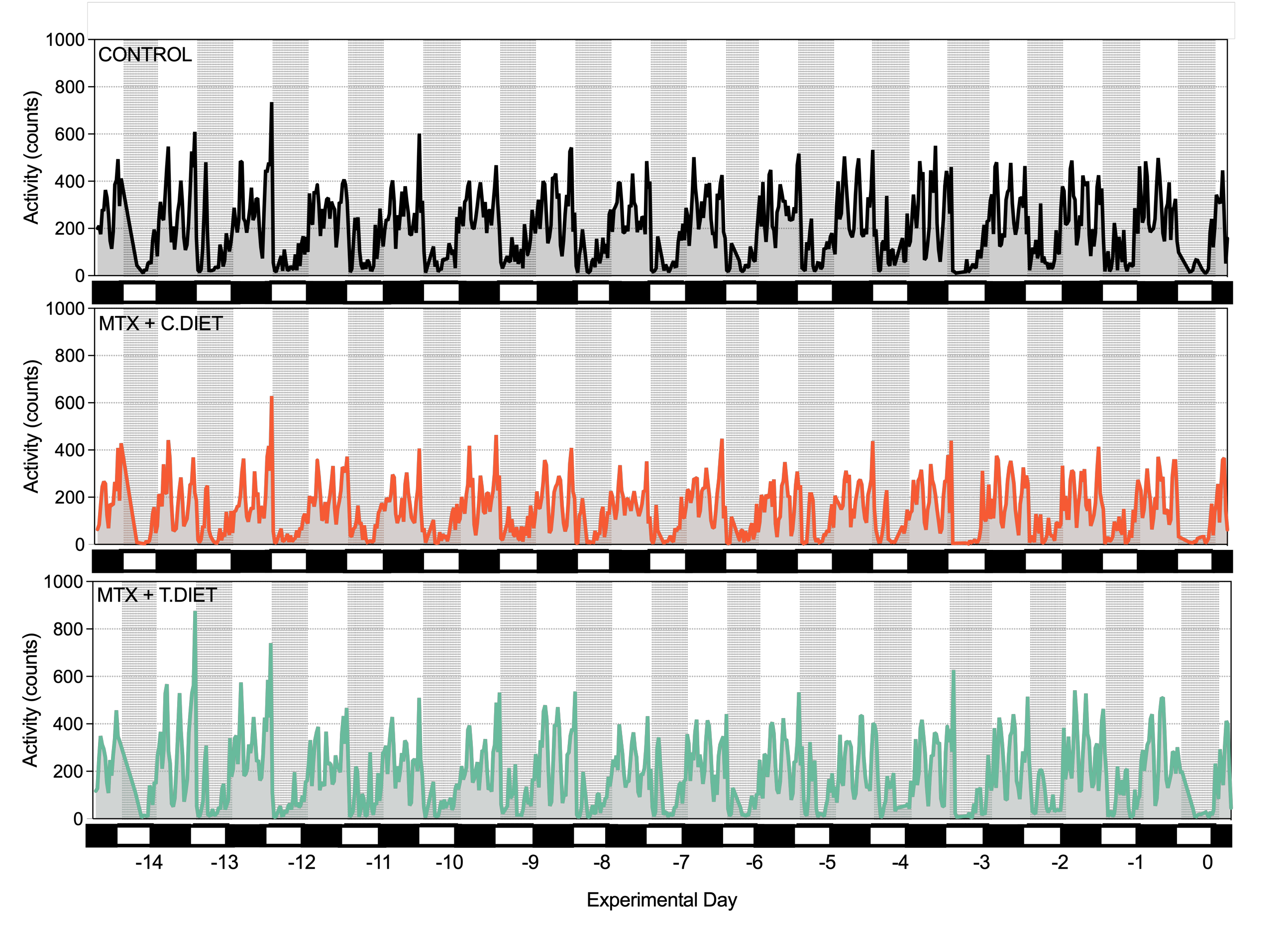

Supplement: Supplementary file 2 — S2 [file 41419_2023_5850_MOESM2_ESM.tif]

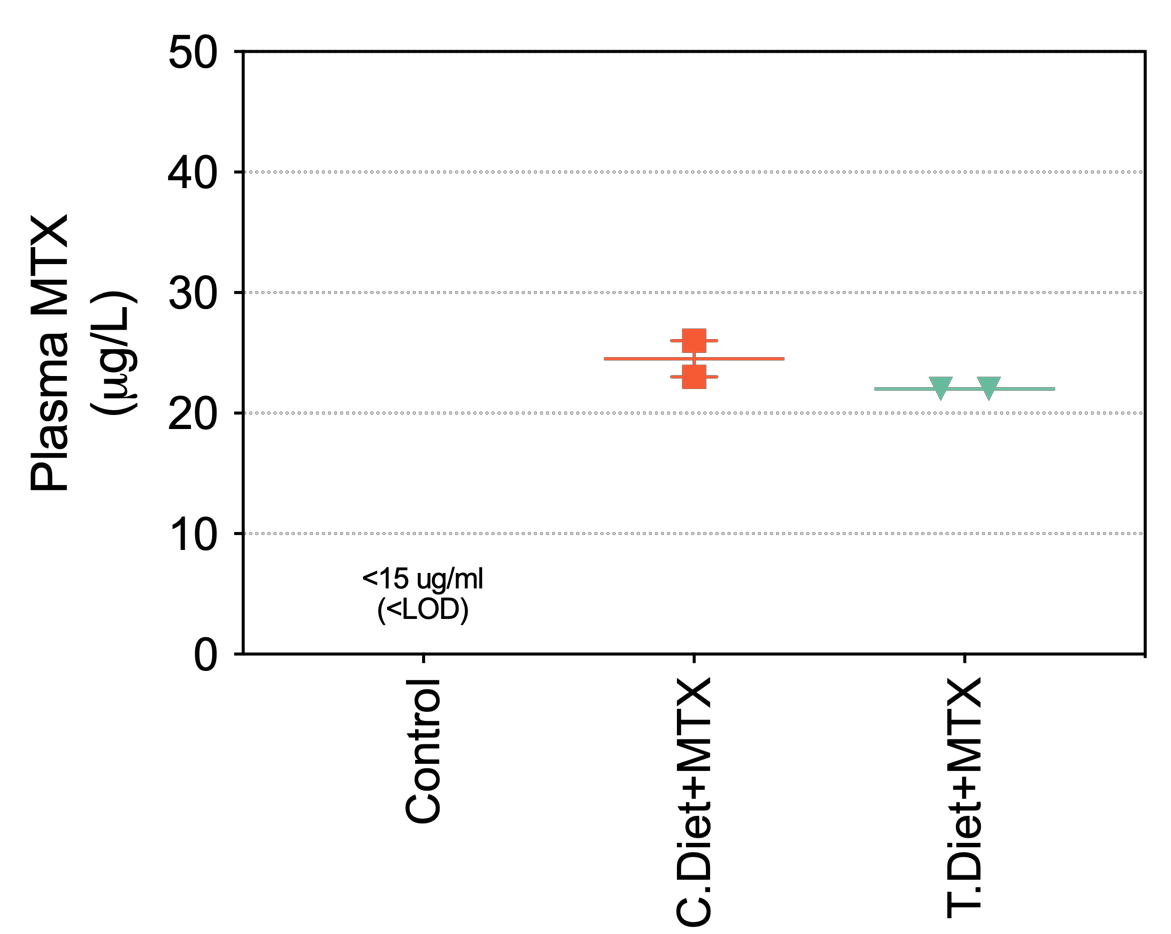

Supplement: Supplementary file 3 — S3 [file 41419_2023_5850_MOESM3_ESM.tif]

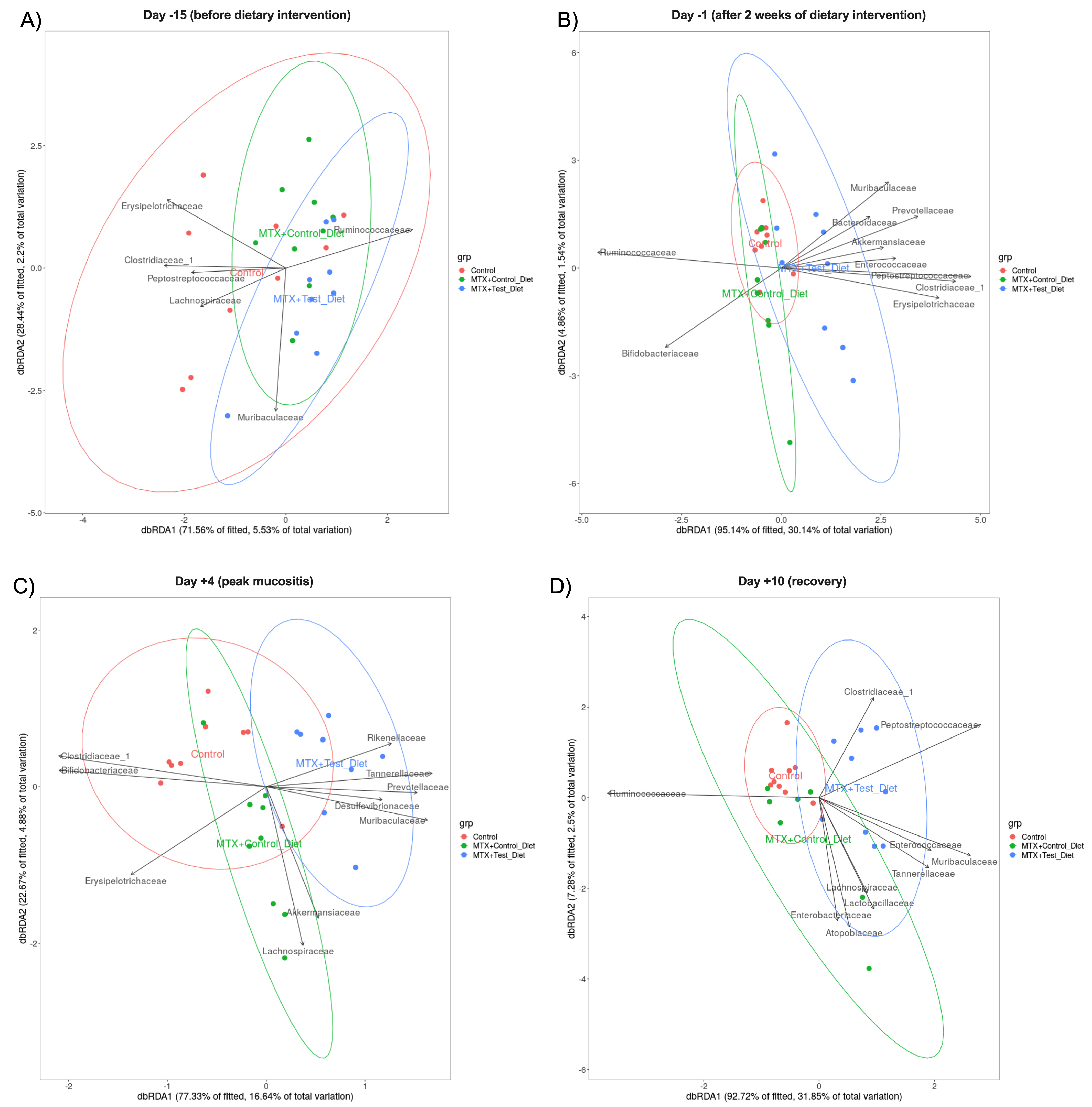

Supplement: Supplementary file 4 — S4 [file 41419_2023_5850_MOESM4_ESM.png]

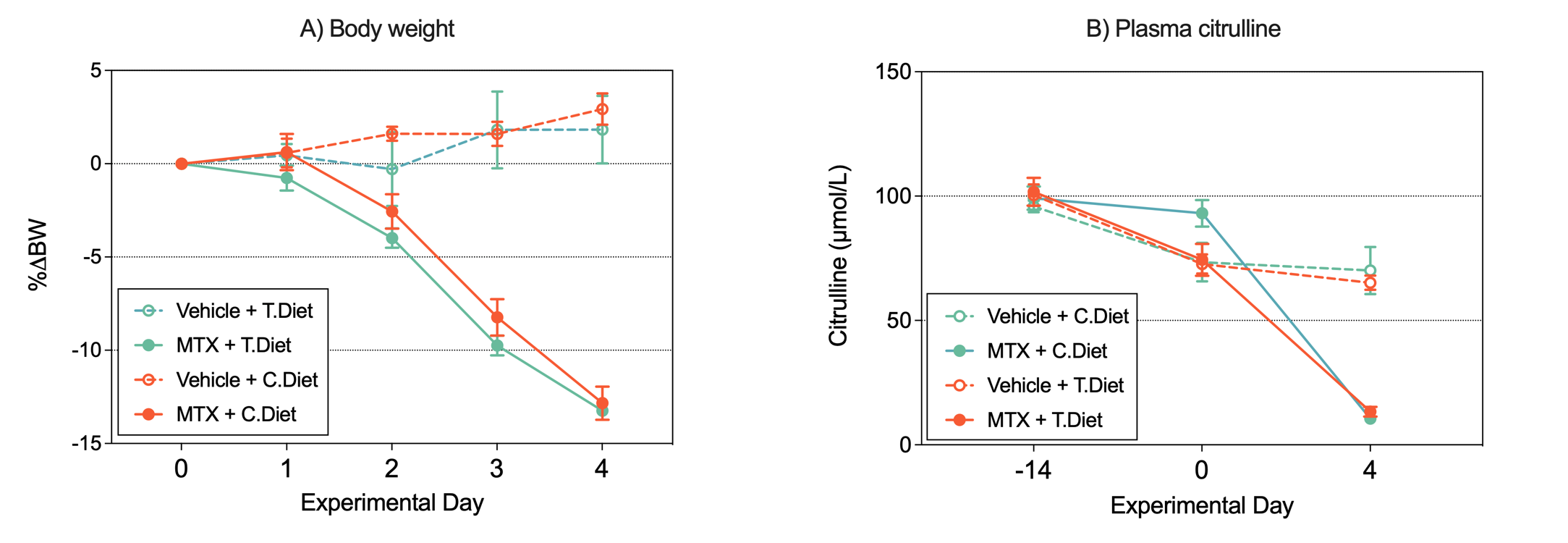

Supplement: Supplementary file 5 — S5 [file 41419_2023_5850_MOESM5_ESM.tif]
