# Supplementary material for: Whey-based diet containing medium chain triglycerides modulates the gut microbiota and protects the intestinal mucosa from chemotherapy while maintaining therapy efficacy
Source: Cell Death Dis. 2023 May 23;14(5):338. doi: 10.1038/s41419-023-05850-9 (PMC10206084; doi:10.1038/s41419-023-05850-9)
Supplement: Supplementary file 6 — Supplementary information [file 41419_2023_5850_MOESM6_ESM.docx]

# Supplementary Information

## Methodology

### Animal ethics and husbandry details with respect to the ARRIVE guidelines

Tumor-naïve studies were conducted in accordance with EU-guidelines for the use of animals for scientific purposes (2010/63/EU), approved by the national Dutch Competent Authority, “Centrale Commissie Dierproeven” (CCD), and the Animal Welfare Body Care of the University Medical Centre Groningen (UMCG), University of Groningen (RUG). All rats were individually housed (to allow for activity, food/water intake monitoring) at the “Centrale Dienst Proefdieren” (CDP, Central Animal Facility) at UMCG. All experiments were performed in N=9 rats which was determined to sufficiently detect an effect size of 25% in the primary outcome measure (alpha=0.05, beta=0.9).

Tumor-bearing studies were conducted in accordance with ethical guidelines approved by the University of Adelaide Animal Ethics Committee in accordance with the National Health and Medical Research Council’s Code for the care and use of animals for scientific research. All experiments were performed with N=8 rats per group deemed to be sufficient to detect an effect size of 25% in the primary outcome measure (alpha=0.05, beta=0.9).

In both cases, rats were individually-housed (to allow food/water intake to be accurately monitored) in conventional, open top cages under 12 h light/dark cycles with *ad libitum* access to food (Table S1) and water (autoclaved tap water). Sawdust bedding was provided in all cases with enrichment (crinkle paper for tumor-naïve model or autoclaved toilet rolls for tumor-bearing model). All cages were randomly arranged across racks to prevent potential bias and dampen variation. All experimental procedures were performed in light phase. All rats were assigned a number at random (generated through random number generator) which was assigned to a treatment group. All procedures/assessments were conducted in a blinded manner (HRW/ARSDF prepared rats/randomisations/performed assessments, RH performed injections), ensuring procedures and biospecimen sampling were randomly allocated with respect to time of day.

# Data


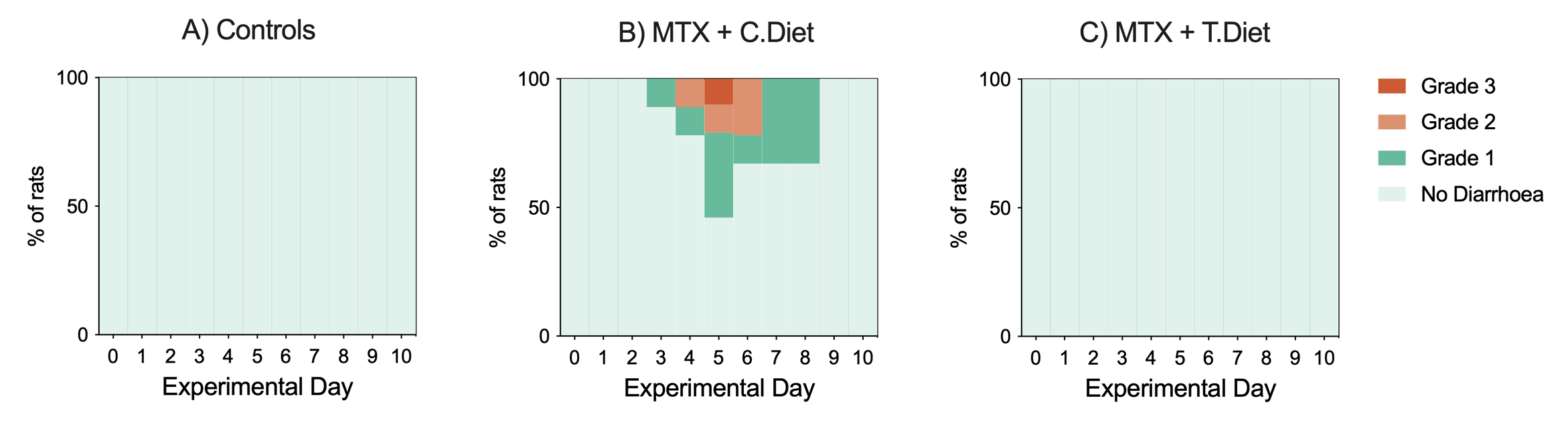


***Figure S1 - MTX-induced diarrhea is prevented by the test diet.*** *Data represent the percentage of rats experiencing varying grades of diarrhea at each experimental time point. Methotrexate caused diarrhea ranging from Grade 1 to Grade 3. No diarrhea was observed when the test diet was administered. Data were compared using a Chi Squared test (P<0.0001).*


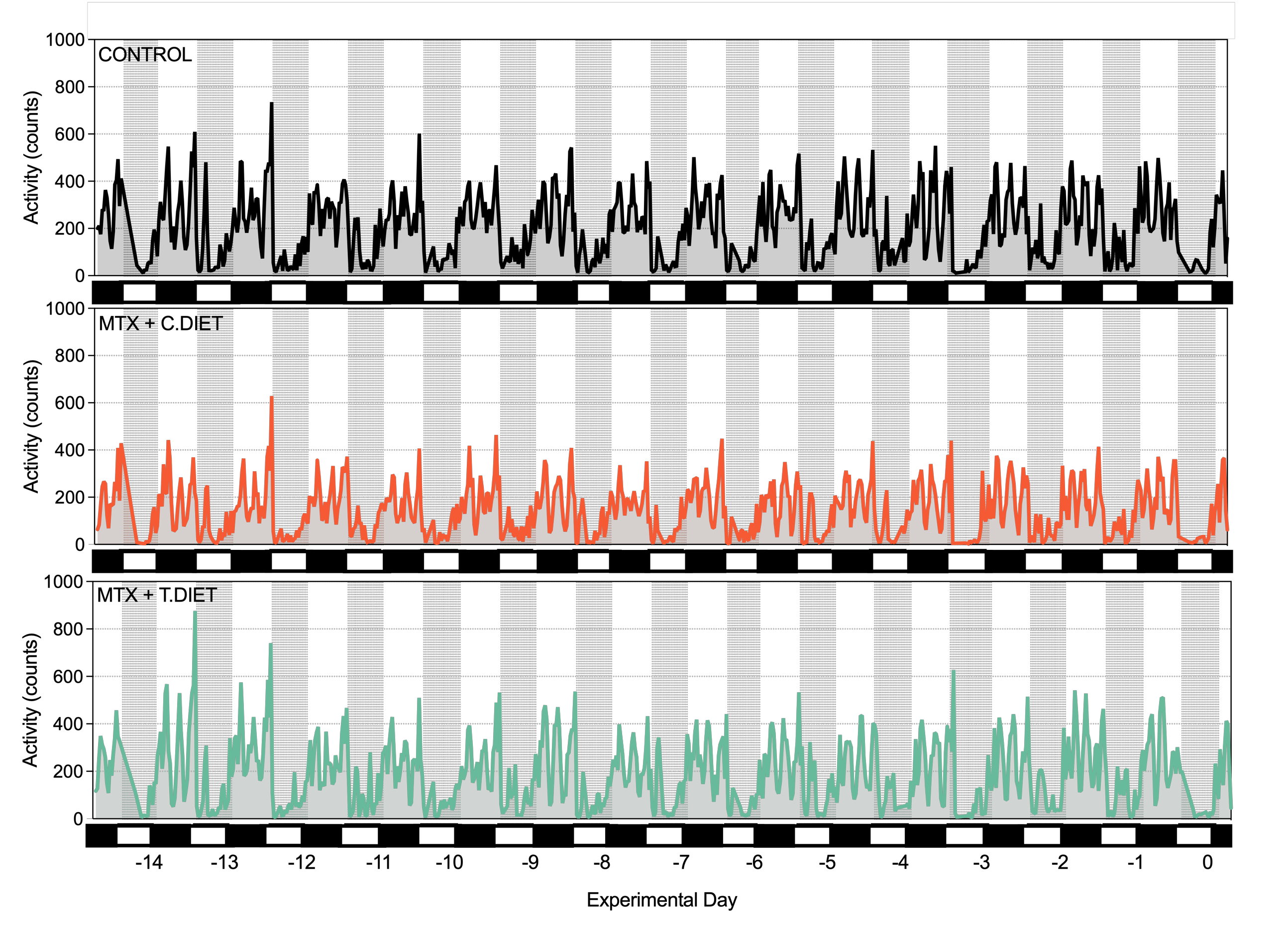


**Figure S2:** Day and night activity in the pre-MTX (dietary intervention) period.


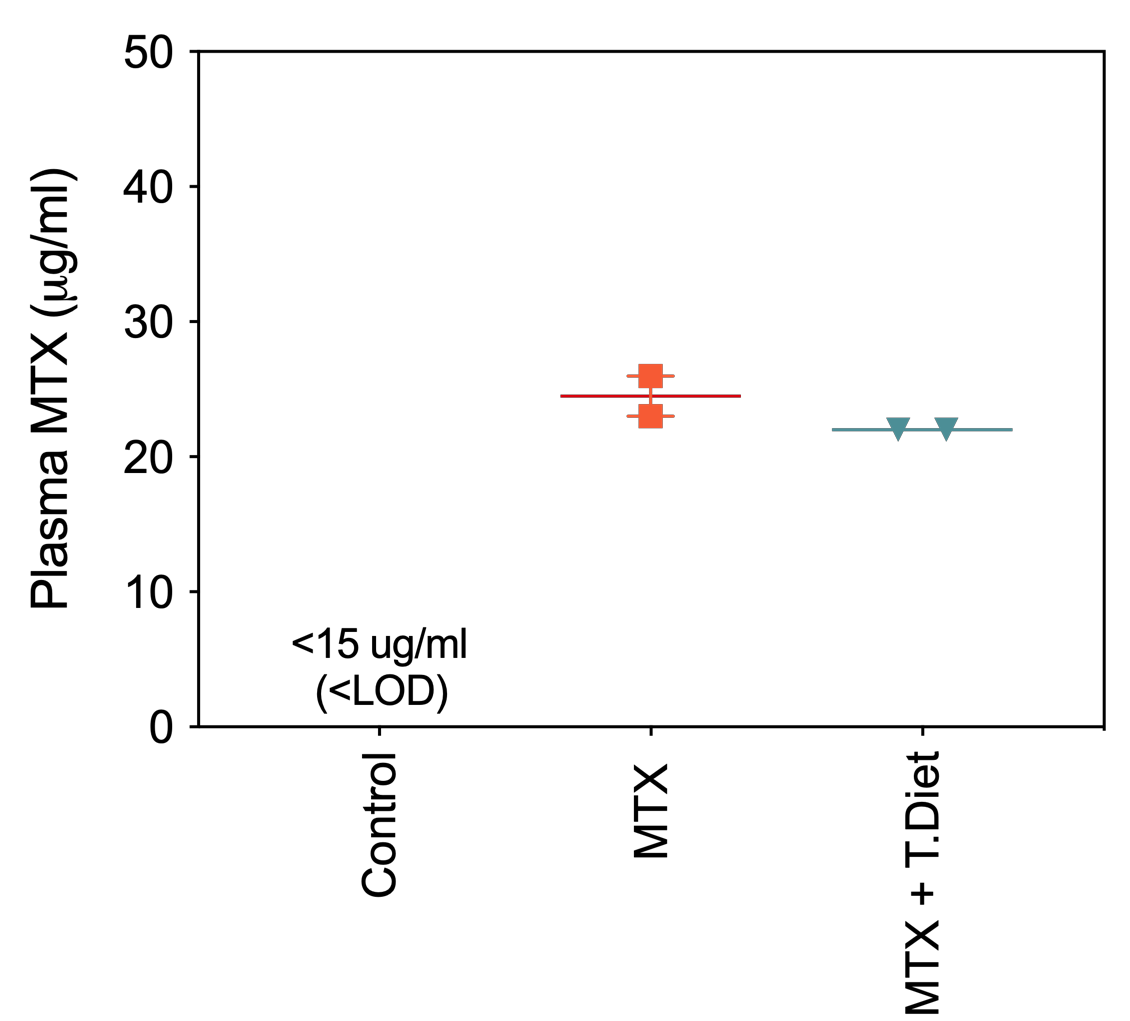


**Figure S3**: Plasma MTX concentrations determined by mass spectrometry in pooled plasma collected 48 hours after administration.


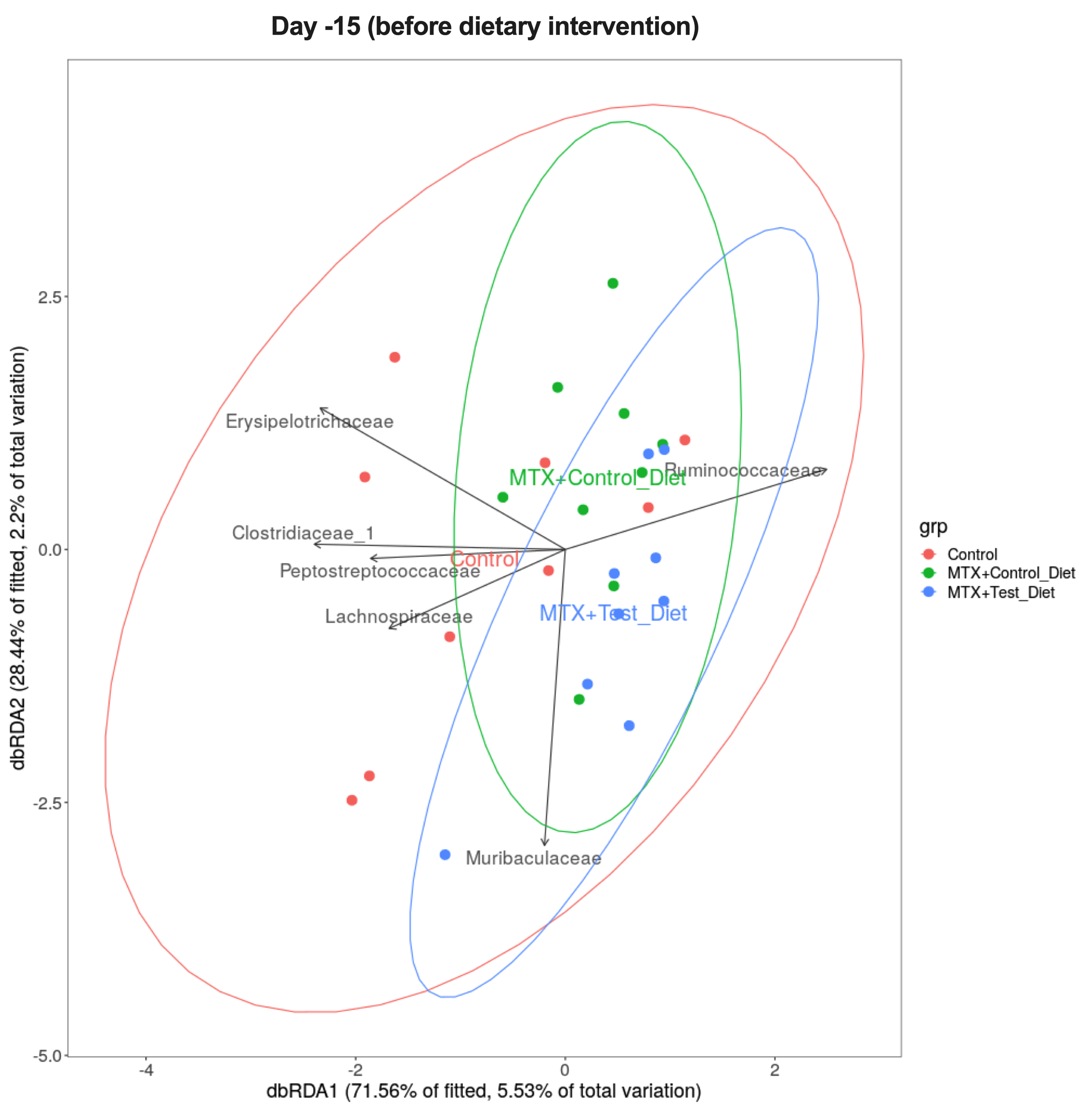


**Figure S4A:** Gut microbiome composition (beta-diversity) at baseline (day -15, prior to intervention)


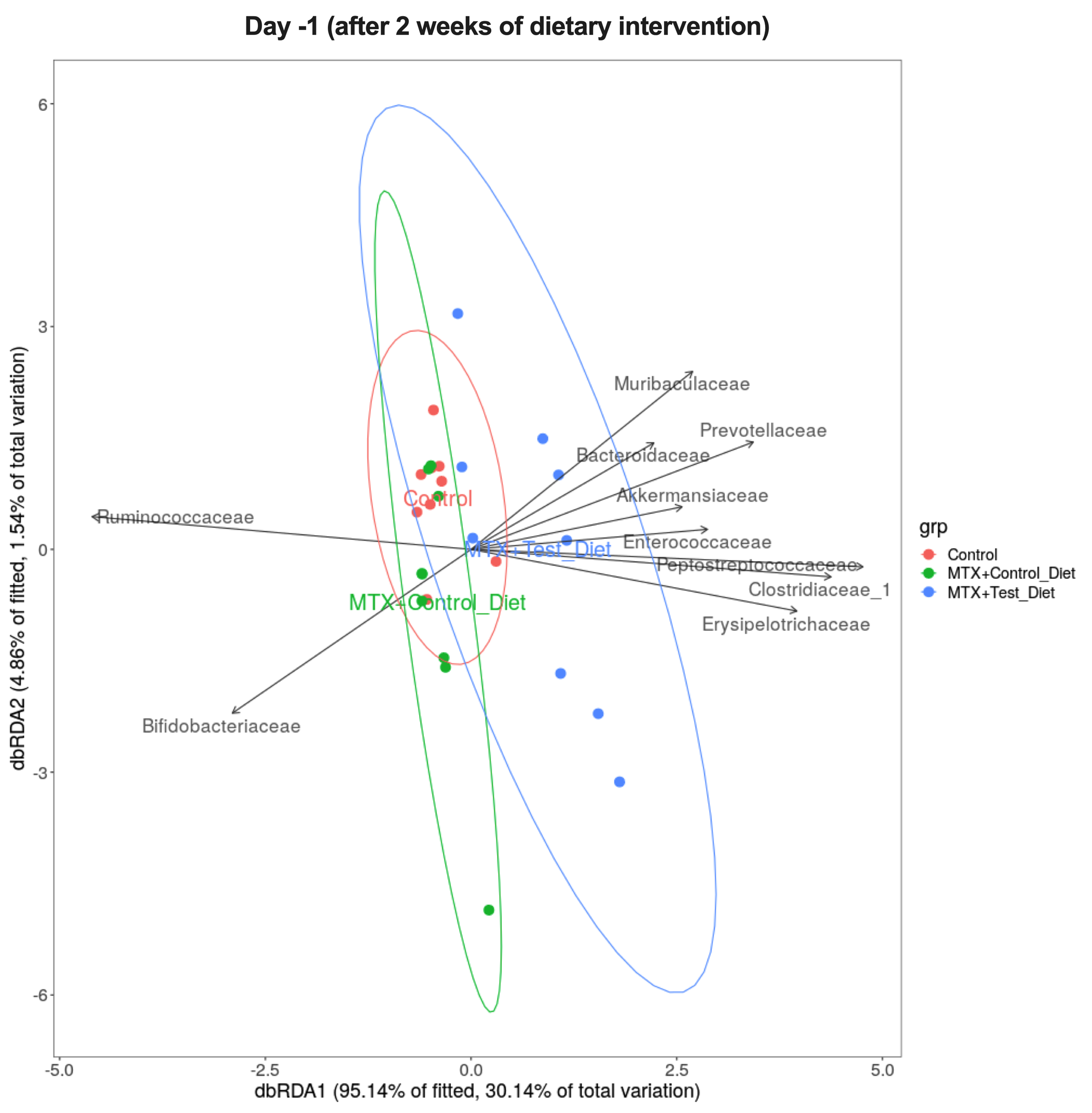


**Figure S4B:** Gut microbiome composition (beta-diversity) at day -1 (prior to MTX)


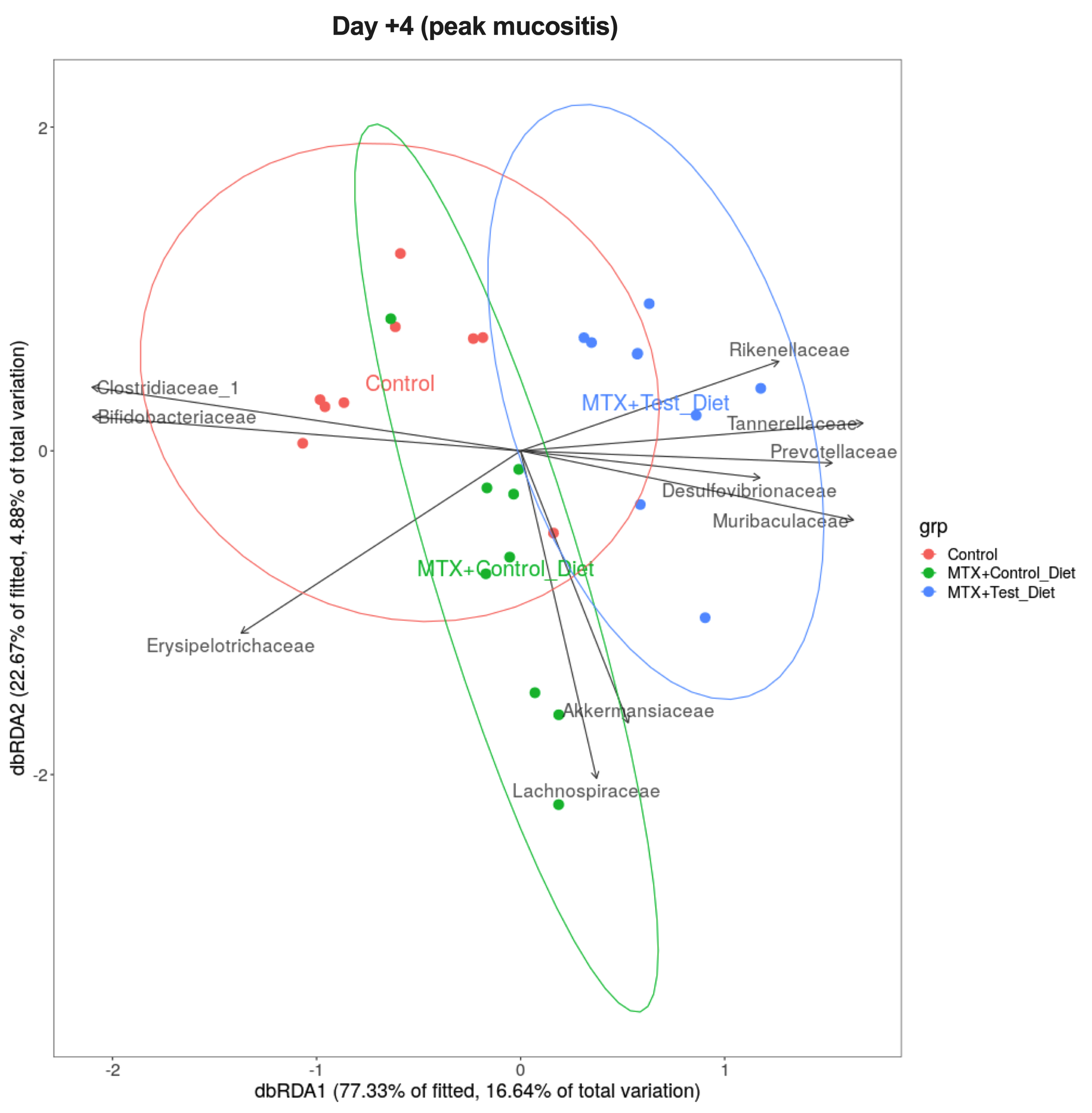


**Figure S4C:** Gut microbiome composition (beta-diversity) at day 4 (peak mucositis)


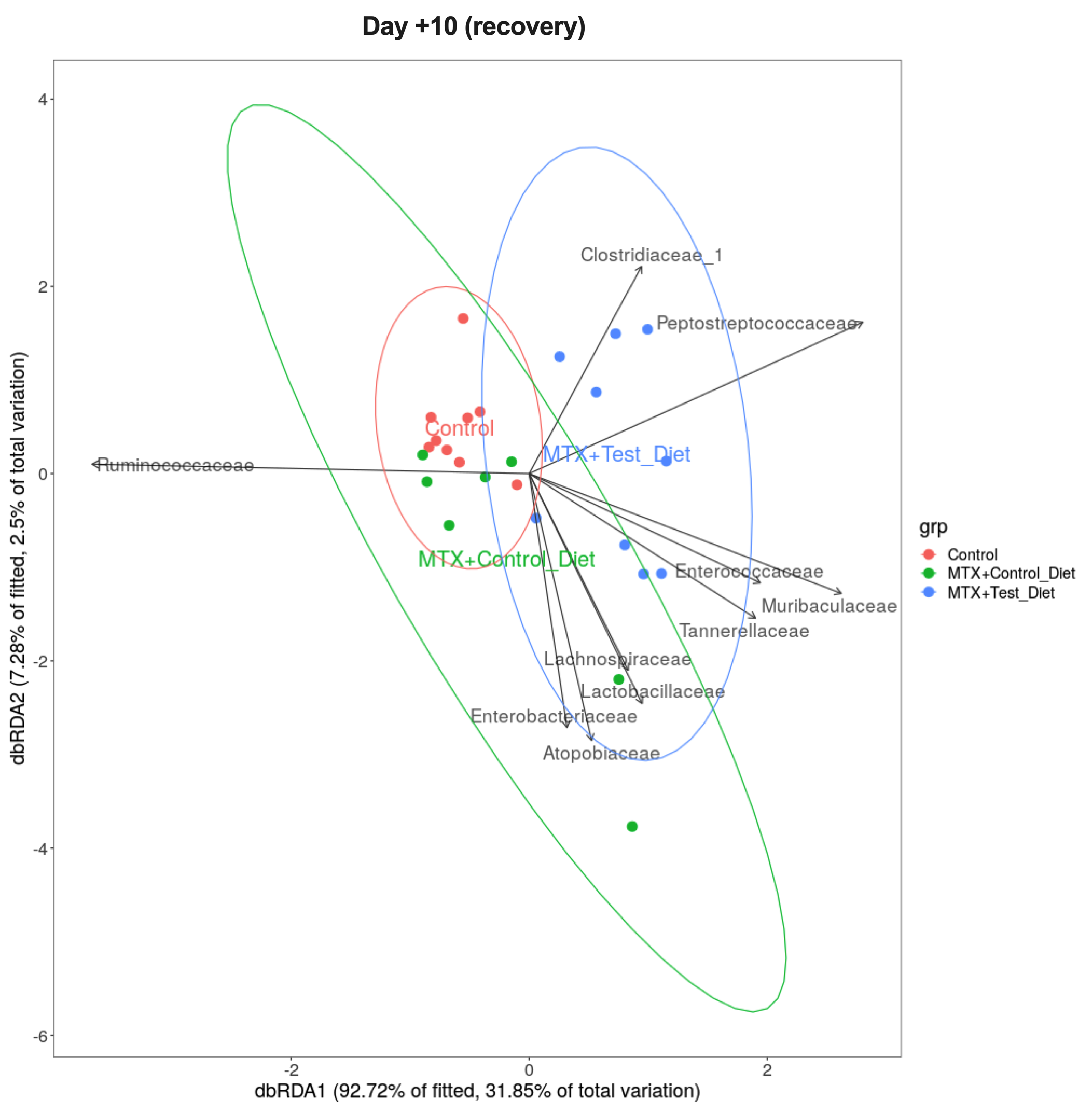


**Figure S4D:** Gut microbiome composition (beta-diversity) at day 10 (mucositis recovery).


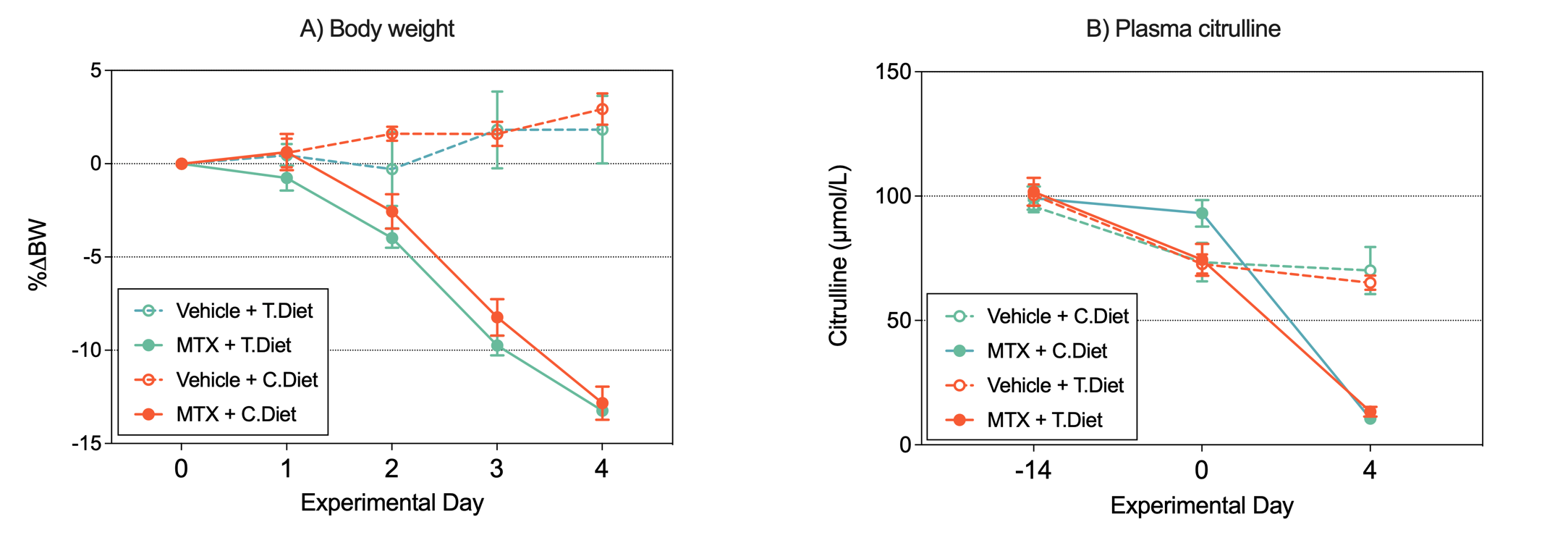


**Figure S5:** Body weight and plasma citrulline in rats from the tumor-bearing model.

| **Table S1: Dietary composition of control and test diets** | | |
| --- | --- | --- |
| **Ingredients (g/kg)** | **Control** | **Test** |
| **Carbohydrates** |  |  |
| Cornstarch | 393.40 | 250.00 |
| Dextrinized cornstarch | 132.70 | 104.50 |
| Sucrose | 100.50 | 100.00 |
| **Fiber** |  |  |
| Cellulose | 47.80 | 40.00 |
| **Protein** |  |  |
| Casein | 201.10 | 0.00 |
| Hydrolyzed Whey | 0.00 | 232.10 |
| **Fat** |  |  |
| MCT | 0.00 | 47.30* |
| Soybean oil | 70.36 | 14.60 |
| **Other** |  |  |
| Mineral Mix | 35.00 | 35.00 |
| Vitamin Mix | 10.00 | 10.00 |
| Choline bitartrate | 2.50 | 2.50 |
| tBHQ | 0.014 | 0.014 |
| **Total Nutritonal value** |  |  |
| Total carbohydrates | 626.60 | 614.40 |
| Total intact protein | 201.30 | 235.70 |
| Total fat | 70.40 | 61.90 |
| **Other specifications** |  |  |
| Kcal | 3821 | 3185 |
| Total weight (g) | 1000 | 993 |
| Protein energy % (EN%) | 22.40 | 22.80 |

*MCT, medium chain triglycerides; tBHQ, tert-butylhydroquinone. Diets are produced as isocaloric.* Please see Table S2 for full breakdown of MCT compositions.*

| **Table S2: Detailed MCT composition** | | | | | | | |
| --- | --- | --- | --- | --- | --- | --- | --- |
| ***Fatty acids (/100 g)*** | | | | | | | |
|  | **Unit** | **Min** | | **Target** | | **Max** | |
| **C8:0 Caprylic acid** | g |  | | 55.2 | |  | |
| **C10:0 Capric acid** | g |  | | 36.8 | |  | |
| ***Fatty acids (/100 g fatty acids)*** | | | | | | | |
| **C6:0 Caproic acid** | g/100g FA | |  | | 0.7* | | 2.0 |
| **C8:0 Caprylic acid** | g/100g FA | | 50.0 | | 60.0* | | 65.0 |
| **C10:0 Capric acid** | g/100g FA | | 34.0 | | 40.0* | | 46.0 |
| **C12:0 Lauric acid** | g/100g FA | |  | | 0.2* | | 1.5 |
| **C14:0 Myristic acid** | g/100g FA | |  | | 0.1* | | 1.0 |
| **C8:0+C10:0** | g/100g FA | | 95.0* | |  | |  |
| **Sum of fatty acid with C≥16** | g/100g FA | |  | |  | | 1.0 |
| **Saturated fatty acids** | g/100g FA | |  | | 100.0 | |  |
| **Trans fatty acids** | g/100g FA | |  | | 0.31 | | 1.0 |

** Indicates certified amount*

| **Table S3: Baseline characteristics (mean, SD)** | | | | | | | |
| --- | --- | --- | --- | --- | --- | --- | --- |
| ***Tumor-naïve model*** | | | | | | | |
|  | | **Control** | | **MTX + C.Diet** | | **MTX + T.Diet** | |
| **Body weight** | | 258.18, 10.74 | | 252.30, 14.85 | | 257.98, 17.27 | |
| **Citrulline** | | 78.42, 11.72 | | 82.40, 10.462 | | 85.65, 7.80 | |
| ***Tumor-bearing model*** | | | | | | | |
|  | **Vehicle + C.Diet** | | **MTX + C.Diet** | | **Vehicle + T.Diet** | | **MTX + T.Diet** |
| **Body weight** | 150.00, 4.75 | | 153.37, 10.45 | | 156.00, 7.96 | | 155.00, 6.61 |
| **Citrulline** | 95.85, 6.61 | | 99.20, 12.43 | | 100.40, 10.53 | | 101.75, 15.86 |
